# An Adaptive Single-Well Stochastic Resonance Algorithm Applied to Trace Analysis of Clenbuterol in Human Urine

**DOI:** 10.3390/molecules17021929

**Published:** 2012-02-15

**Authors:** Wei Wang, Suyun Xiang, Shaofei Xie, Bingren Xiang

**Affiliations:** 1 Department of Analytical Chemistry, China Pharmaceutical University, Nanjing 211198, China; 2 Jiangsu Key Laboratory for Supramolecular Medicinal Materials and Applications, College of Life Science, Nanjing Normal University, Nanjing 210046, China; 3 Nanjing Changao Pharmaceutical Technology Limited, Nanjing 210022, China; 4 Key Laboratory of Drug Quality Control and Pharmacovigilance (Ministry of Education), China Pharmaceutical University, Nanjing 210009, China

**Keywords:** adaptive stochastic resonance algorithm, single-well, genetic algorithm, trace analysis, clenbuterol

## Abstract

Based on the theory of stochastic resonance, an adaptive single-well stochastic resonance (ASSR) coupled with genetic algorithm was developed to enhance the signal-to-noise ratio of weak chromatographic signals. In conventional stochastic resonance algorithm, there are two or more parameters needed to be optimized and the proper parameters values were obtained by a universal searching within a given range. In the developed ASSR, the optimization of system parameter was simplified and automatic implemented. The ASSR was applied to the trace analysis of clenbuterol in human urine and it helped to significantly improve the limit of detection and limit of quantification of clenbuterol. Good linearity, precision and accuracy of the proposed method ensure that it could be an effective tool for trace analysis and the improvement of detective sensibility of current detectors.

## 1. Introduction

Trace analysis is one of the routine tasks for analytical chemists in various fields of analytical chemistry. Highly sensitive detection techniques are helpful to reduce workload and enhance analysis accuracy. To pursue detection sensitivity, one approach is via hardware improvements, whereas an alternative approach is to enhance the signal-to-noise ratio (SNR) by chemometric methods. Various smoothing and filtering methods are widely used to improve the signal of interest by reducing the effect of noise [1,2,3,4]. However, they may result in loss (hopefully negligible) of useful information. Different from these methods, a stochastic resonance (SR) algorithm can amplify the weak signal significantly in a nonlinear system by making use of noise instead of filtering it [5,6,7]. Energy transfer from noise to useful signals will take place when signal and noise cooperate properly within the nonlinear system, *i.e.*, the SR condition is reached, and accordingly, the SNR of useful signals will be enhanced. 

For superior detection of useful signal submerged in heavy noise, the SR algorithm has received increasing interest from analytical chemists and has been successfully applied in pharmaceutical analysis [8,9], food analysis [10,11] and environmental analysis [12,13]. In the algorithm, a nonlinear system is one of the necessary factors for SR. The most frequently used nonlinear system in conventional SR is a bistable system described as a double-well potential. However, in the double-well potential stochastic resonance (DSR) system, two parameters named *a* and *b* are involved and need to be optimized for SR. In order to obtain an ideal output of the algorithm, additional parameters are often introduced into the system [14,15], so there are two or more parameters and the optimizations are somewhat complicated. In 1993, Stocks replaced the bistable system with a monostable one with only one parameter [16]; and later, Zhang *et al.* developed a single-well potential stochastic resonance (SSR) with only one parameter to simply the algorithm [17].

The optimization of system parameters is essential for the application of the algorithm of SR. While in the previous studies, the optimal parameters were achieved by a universal one by one search within a given range, sometimes this was complicated and time consuming. A genetic algorithm (GA) is an adaptive heuristic search algorithm premised on the evolutionary ideas of natural selection and genetic [18,19,20]. It is a particular class of evolutionary algorithms that use techniques inspired by evolutionary biology such as inheritance, mutation, selection, and crossover. Being a population-based approach, GA is well suited to implement the automatic optimization of system parameters of the SR algorithm. 

Clenbuterol is a selective adrenoceptor selective agonist and its main therapeutic use is in the treatment of pulmonary diseases for its longtime bronchodilator activity. Due to its similar physiological effects to sympathomimetic and anabolic steroids, the drug is popularly abused as a doping agent by athletes to increase performance, especially in bodybuilding, power-related and endurance sports. The abuse of clenbuterol hurts fair competition and athlete’s health, so accordingly its use is prohibited both in and out of competition by the International Olympic Committee [21]. However, the use of masking agents, such as diuretics, makes the detection more difficult, so, a highly sensitive and reliable assay method is essential. Various methods have been used in the determination of clenbuterol in biological matrices. The reported methods include enzyme-linked immunosorbent assay (ELISA) [22,23,24], gas chromatography-mass spectrometry (GC/MS) [25,26], liquid chromatography-tandem mass spectrometry (LC/MS/MS) [27,28] and hapten microarrays [29]. Among these methods, some lack specificity for the possible cross-reactivity, some are time-consuming due to complicated pretreatments and some need advanced detectors or special devices. The aim of the present work was to develop an adaptive single-well potential stochastic resonance algorithm (ASSR) coupled with GA for the trace analysis of clenbuterol in urine as a biological matrix. By applying the algorithm, the weak signal of clenbuterol is amplified significantly and those buried in noise could be determined accurately. The detection sensitivity is comparable or better than that of reported methods that require advanced detectors, and it may be expected that those advanced detectors would be more made more sensitive by the application of the proposed algorithm.

## 2. Theory and Algorithm

### 2.1. Theory of Single-Well Potential Stochastic Resonance

The nonlinear Langevin equation has often been used to describe the SR phenomenon. It has the following expression [30]:

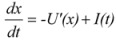
(1)
where the variable *x* is the output of the nonlinear system, representing the position of signal. *I(t)* = *S(t)* + *N(t)* denotes an input signal embedded in a noisy environment. *S(t)* is the signal and *N(t)* is the noise. 

The single-well potential system in the algorithm of SSR developed by Zhang *et al.* [17] can be described by the following equation, where there’s only one parameter *b* defining the potential profile: 


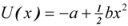
(3)

**Figure 1:**
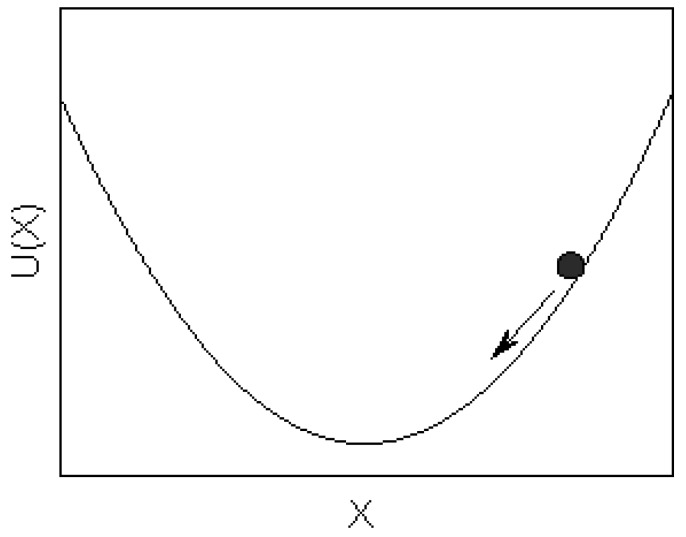
A typical single-well potential.

As shown in Figure 1, the single-well potential has no potential barrier. When SR takes place, the input signal will extract energy from the noise to move along the brim of the single-well potential. Thus, it can achieve a height that the single input signal cannot reach, and for this reason the strength of signals will increase and that of noise will decrease. Consequently, the output of a weak signal will give a better SNR than its input.

In the proposed SSR, the discrete stochastic differential equation [obtained by substitution of Eqution(3) into Eqution(1)] was solved by a fourth-order Runge-Kutta method [17]. The calculation procedure starts with normalization of the input signal *I(t)* to the interval [–1,1], and the normalized signal is then operated on by the algorithm to give the output signal. The final results can be obtained by inverse normalization of the output signals. 

### 2.2. Genetic Algorithm

When the algorithm of SSR applied to quantitative determination, the optimization of *b* is essential for this parameter will affect the profile of the potential well and the final output results. In this work, the parameter *b* was optimized to give out a maximal SNR of the output signal. Here, the objective function of the SSR model can be defined as follows:

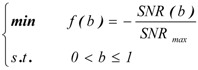
(4)
where *SNR(b)* is the SNR of system output signal and *SNR_max_* is the maximal SNR with a different parameter *b*.

GA is a commonly used approach to solving optimization problems. Here, vector evaluated GA (or VEGA) proposed by Schaffer [31] was used to implement the automatic optimization of the parameter *b*. In VEGA, a population *p_t_* is randomly divided into K equal sized subpopulations:*p*_1_, *p*_2_, …, *p*_K_. Then, each solution in subpopulation *p_i_* is assigned a fitness value based on objective function *z_i_*. Solutions are selected from these subpopulations using proportional selection for crossover and mutation. Crossover and mutation are performed on the new population in the same way.

In this paper, the parameter *b* of SSR was optimized by VEGA. The procedures of optimization are as follows:

*N_s_* = subpopulation size (*N_s_* = *N*/*K*).
Step 1: Start with a random initial population *P_0_*. Set *t* = *0*.
Step 2: If the stopping criterion is satisfied, return *P_t_*.
Step 3: Randomly sort population *P_t_*.
Step 4: For each objective *k*, *k* = 1,…, K, perform the following steps:
Step 4.1: For *i* = *1* + *(k−1)N_s_*,…,*kN_s_*, assign fitness value *f(x_i_)* = *z_k_(x_i_)* to the *i* th solution in the sorted population.
Step 4.2: Based on the fitness values assigned in Step 4.1, select *N_s_* solutions between the (*1* + *(k−1)N_s_*)th and (*kN_s_*)th solutions of the sorted population to create subpopulation *P_k_*.
Step 5: Combine all subpopulations *P_1_*,…, *P_k_* and apply crossover and mutation on the combined population to create *P_t_*_+1_ of size *N*. Set *t* = *t* + *1*, go to Step 2.
The parameters used in implementing GA are as follows:
Number of individuals: 200;
Maximum number of generations: 100;
Precision of variables: 20;
Generation gap: 0.7;

All the calculations were performed using programs written by the authors in the Matlab 7.0 environment (Mathworks, Natick, MA, USA), running on a PC with an Intel(R) Core Duo CPU 1.83 GHz and 1G RAM.

## 3. Results and Discussion

### 3.1. Optimization of System Parameter

When the input signal is fixed, the nonlinear system parameters will directly influence the quality of final output signal and consequently influence the results of quantitative determination. A raw chromatogram of clenbuterol at a concentration of 0.2 ng/mL with an original SNR of 6.1was used for the optimization. As a result of optimization, a maximal SNR of 22.6 was obtained when *b* = 0.0574.

### 3.2. Quantitative Analysis of Clenbuterol in Urine

Although the raw intensities of clenbuterol vary with the spiked concentrations in target samples, the same parameter will be used throughout to keep the quantitative relationship of the output signals. A set of spiked urine samples with clenbuterol at different concentration levels were prepared and assayed, and then the chromatograms were processed by ASSR with the optimized parameter (*b* = 0.0574) developed above. 

**Figure 2:**
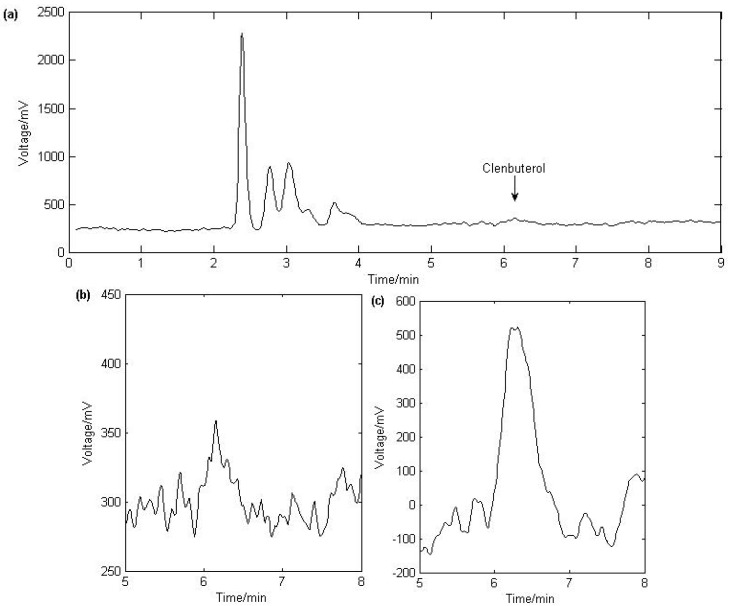
Chromatogram of urine spiked with clenbuterol at 0.025 ng/mL. (**a**) Full length of the chromatogram. (**b**) Especially enlarged chromatogram of clenbuterol. (**c**) The peak of clenbuterol obtained by ASSR with *b* = 0.0574.

The specificity of the assay was studied by using blank urine samples. No endogenous interferences were observed both before and after applying the algorithm. Signal-to-noise ratio of 3 and 10 are usually considered as limit of detection (LOD) and limit of quantification (LOQ), respectively. The LOD and LOQ of LC-MS analysis of clenbuterol can be estimated at 0.1 ng/mL and 0.3 ng/mL, respectively. With the application of ASSR, the LOD and LOQ were respectively improved to 0.025 ng/mL and 0.05 ng/mL. The raw chromatogram of LOD is shown in Figure 2(a) and (b) wherein the clenbuterol peak is buried in noise. It is obvious that the weak signal is hard to detect. After being processed by ASSR, the output signal of clenbuterol showed a significant SNR improvement [shown in Figure 2c].

**Figure 3:**
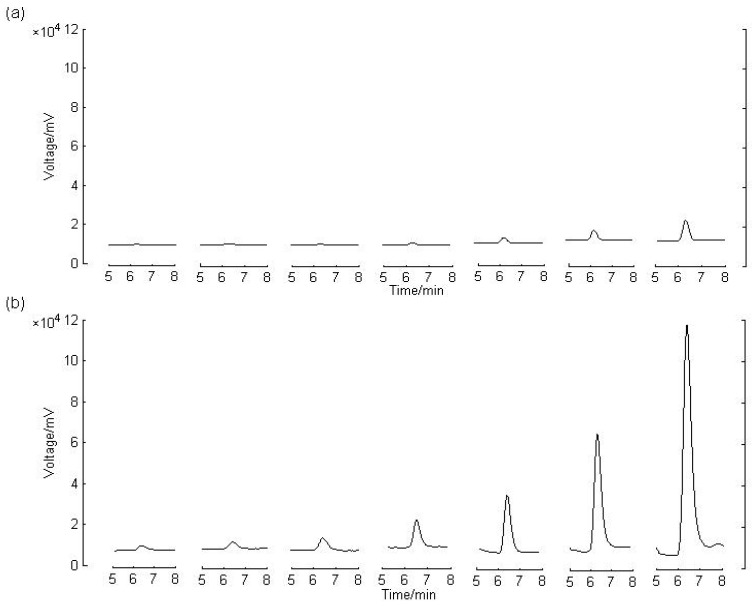
Chromatograms of clebuterol in urine samples of calibration curve. (**a**) The raw chromatograms of clenbuterol. (**b**) The chromatograms of clenbuterol obtained by ASSR with *b* = 0.0574. From left to right, the concentrations of clenbuterol are 0.2, 0.5, 1.0, 2.0, 5.0, 10.0 and 20.0 ng/mL, respectively.

The calibration curve was prepared and assayed with the urine samples spiked with clenbuterol at concentrations ranging from 0.2 ng/mL to 20.0 ng/mL. Then the raw chromatograms of clenbuterol were processed with the optimized ASSR. A comparison is made between the raw chromatograms and those obtained by ASSR in Figure 3.

The calculated curve illustrated in Table 1 displays a good linear relationship between the peak area ratio (*f* ) of clenbuterol to IS and clenbuterol concentration. The results show that all the original signals of target analyte are significantly enhanced and maintain a good linear relationship as well.

**Table 1:** Calibration curve of clenbuterol by ASSR.

Conc. (ng/mL)	0.2	0.5	1	2	5	10	20
Ratio( *f* )	0.243	0.465	0.917	1.689	3.311	6.918	14.716
Calibration Curve	*f* = 0.722C + 0.0453 r = 0.999						

The investigation of precision and accuracy was carried out with spiked preparations at concentration of 0.5, 2.0 and 10.0 ng/mL. Intra-day precision was determined by repeated analysis of urine samples within one day (n = 6), and inter-day precision was determined by repeated analysis of urine samples on three consecutive days (n = 6 series per day). The method accuracy was testing from recovery analysis which was defined as the ratio of the measured concentration to the added concentration. The intra- and inter-day assays are summarized in Table 2 and the results indicate that the accuracy and precision of the assay are acceptable.

**Table 2:** Acurracy and precision of clenbuterol by ASSR.

Added Conc.	Intra-day assay			Inter-day assay		
	Measured Conc.	Precision	Accuracy	Measured Conc.	Precision	Accuracy
(ng/mL)	(mean ± S.D.) (ng/mL)	R.S.D (%)	(%)	(mean ± S.D.) (ng/mL)	R.S.D (%)	(%)
0.5	0.55 ± 0.04	7.6	109.5	0.56 ± 0.05	8.7	111.9
2.0	2.07 ± 0.12	5.8	103.5	1.87 ± 0.14	7.3	93.4
10.0	9.60 ± 0.53	5.5	96.0	9.87 ± 0.55	5.6	98.7

## 4. Experimental

### 4.1. Materials and Reagents

Standards of clenbuterol and terazosin (used as internal standard) were obtained from the National Institute for the Control of Pharmaceutical and Biological Products (Beijing, China). HPLC grade methanol and acetonitrile were purchased from Merck (Germany). Acetic acid and ammonium hydroxide, both analytical grade, were obtained from Nanjing Chemical Reagent Co., Ltd. (Nanjing, China). Double distilled water was used throughout the study. SCX SPE (250 mg) and C18 SPE (250 mg) disposable cartridges (Hanbon Sci. & Tech., Jiangsu, China) were used for sample pretreatment and purification. 

### 4.2. Standard Solution

Standard stock solutions at 1.0 mg/mL of clenbuterol and terazosin were prepared in methanol and stored at 4 °C. Working solutions of clenbuterol (10, 100 and 1,000 ng/mL) and terazosin (1,000 ng/mL) were prepared by dilution with methanol.

### 4.3. Sample Preparation

C_18_ SPE cartridge and SCX SPE cartridge fixed in downward mode were conditioned successively with methanol, water and 30 mmol/L hydrochloric acid (5.0 mL each). Human urine (5 mL) spiked with clenbuterol and internal standard (terazosin) was adjusted to about pH 5.3 with acetic acid (200 μL, 1%, v/v), vortex mixed for 5 s and loaded onto the C_18_ cartridge. Then cartridge was washed with water (5 mL) and methanol (5 mL), then the C_18_ cartridge was removed, and the SCX cartridge was eluted with methanol (5 mL, 4% ammonia, v/v). Eluates were evaporated with nitrogen at 50 °C, and the residues were reconstituted with acetonitrile (200 μL).

### 4.4. LC/MS Analysis

The analysis was performed on an HP1100 LC/MSD system (Hewlett-Packard, USA), with the liquid chromatography part consisting of an automatic sampler and a binary pump with an online degasser, and the mass spectrometry unit consisting of a single quadrupole mass spectrometer equipped with an electrospray ionization interface (ESI). The separation was carried on a Diamonsil C_18_ column (250 × 4.6 mm, i.d.; 5 μm, Dikma, China) with temperature set at 25 °C. The mobile phase consisted of 0.02 M ammonium formate buffer (pH 3.5)-acetonitrile (72:28, v/v) at a flow rate of 1.0 mL/min. Mass spectrometric detection was operated in the positive mode with the fragmentor voltage set at 70 V and capillary voltage at 4kV, drying gas (N_2_) flow of 10.5 L/min and temperature of 350 °C, nebulizer pressure of 45 psi. The [M+H]^+^, *m/z* 277 for clenbuterol and [M+H]^+^, *m/z* 388 for terazosin were selected as detection ions, respectively.

## 5. Conclusions

An adaptive single-well stochastic resonance algorithm was developed for trace analysis. In the proposed algorithm, there is only one system parameter and this parameter was automatic optimized by a genetic algorithm. The simplified optimization progress makes the algorithm easier to manipulate in practice. By applying the algorithm, weak signals of clenbuterol were amplified significantly, the LOD and LOQ were improved from 0.1 ng/mL to 0.025 ng/mL and from 0.3 ng/mL to 0.05 ng/mL, respectively. It could be expected those advanced detectors coupled with the proposed algorithm would be more effective for trace analysis.
